# Using Network Analysis to Identify Central Facets of Androgynous Development Between Sexes in Chinese Adolescents

**DOI:** 10.3390/bs15101375

**Published:** 2025-10-09

**Authors:** Xisha Liu, Weijun Liu

**Affiliations:** 1 Mental Health Education Center, Hainan University, Haikou 570228, China; 996221@hainanu.edu.cn; 2 School of Psychology, Hainan Normal University, Haikou 571158, China; 3 Faculty of Psychology, Southwest University, Chongqing 400700, China

**Keywords:** network analysis, androgyny, masculinity, femininity, sex difference

## Abstract

Androgyny, characterized by high levels of both masculinity and femininity traits, is linked to adaptive psychological outcomes. However, existing research has typically examined these traits at the latent variable level, obscuring the specific trait facets that are central to androgynous development. Using network analysis, this study investigated the androgynous structure network at the level of trait facets to identify the most influential facets and explore sex-specific structures. A convenience sample of 1270 Chinese adolescents (*M*_age_ = 15.41, *SD* = 0.88; 611 females) completed the validated Chinese Sex-Role Inventory, which measures 32 facets of masculinity and femininity traits. In the full sample, “calm” exhibited the highest expected influence (EI = 1.11). Crucially, the masculinity facet “magnanimous” was the most powerful bridge to the femininity network (bridge EI = 1.56), particularly for males (bridge EI = 1.18); the femininity facet “thoughtful” (bridge EI = 0.97) was the most powerful bridge to the masculinity network, especially for females (bridge EI = 0.86). Significant sex differences were observed in global EI, with females showing greater global network activation (*p* = 0.008). The sex difference was additionally evident in “thoughtful” (male < female, *p* = 0.022) and “magnanimous” (male > female, *p* = 0.043). Such findings highlight the pivotal roles of “magnanimous” for males and “thoughtful” for females in fostering androgyny. The study advances the understanding of androgyny by delineating its facet-level structure and underscores the value of sex-specific strategies in fostering balanced gender-typed trait development. The convenience sample may limit the generalizability of these findings.

## 1. Introduction

In a classroom setting, a boy who openly expresses anxiety before an exam might be perceived as lacking emotional control, while a girl who confidently voices her opinion in a group discussion could be labeled aggressive. Such everyday examples illustrate how culturally constructed gender-typed traits shape different expectations for emotional expression and social behavior for males and girls.

Gender-typed traits are socially desirable attributes associated with a particular sex (Bem, 1981; W. Liu et al., 2024; Ying et al., 2024). Masculinity and femininity traits represent two primary dimensions of gender-typed traits (Hsu et al., 2021). It is worth noting that masculinity and femininity traits are socially constructed, non-essential, and fluid sets of attributes, behaviors, and roles, rather than fixed or innate properties determined by sex (Olavarría, 2023). Moreover, although traditionally conceptualized as opposite ends of a single continuum, contemporary perspectives posit them as orthogonal dimensions that can coexist within an individual (Bem, 1984). This means that an adolescent, regardless of sex, can possess high levels of both masculinity (e.g., brave) and femininity (e.g., gentle)—a profile known as androgyny (D.-Z. Liu et al., 2011; Spence et al., 1974). Research has linked androgyny to a range of adaptive outcomes, including lower depression (Lin et al., 2021), higher academic performance (Skinner et al., 2019), and greater school-related well-being (Korlat et al., 2022). Given these benefits, fostering androgynous development is an important goal.

A defining characteristic of androgyny is the co-presence of high levels of both masculinity and femininity traits (Safraou et al., 2025; Spence et al., 1974), which implies a positive association between them. For example, a large-scale study of 3808 Chinese adolescents reported a correlation of *r* = 0.64 between masculinity and femininity (W. Liu et al., 2024), a finding echoed in another study of 512 Chinese adolescents, which found correlations of *r* = 0.73 for males and *r* = 0.65 for females (Ying et al., 2024). This pattern extends beyond Chinese samples, with similar positive correlations observed in 354 Turkish undergraduates (*r* = 0.30) (Ozkan & Lajunen, 2005), and 417 German adults (*r* = 0.18–0.50) (Krahe, 2018). Collectively, these studies demonstrate the robustness of the positive masculinity-femininity relationship across diverse cultures and age groups, providing strong evidence for the existence of an androgynous structure at a latent variable level.

However, correlation analyses between masculinity and femininity offer limited insight into the finer-grained organization of the two traits at the facet level (Chen et al., 2025). For instance, the correlation analyses cannot reveal the specific relationship between being brave and being gentle. This limitation represents a critical gap, cause a deeper understanding of these facet-level interconnections is necessary to move beyond simplistic strategies that merely aim to enhance femininity in males or masculinity in females. A more productive approach requires identifying the most influential specific facets within the androgynous structure. Pinpointing these central facets, particularly those that act as bridges between masculinity and femininity, can inform targeted interventions designed to foster androgynous development.

Furthermore, it is important to consider the role of sex when examining these core facets, as sex differences in masculinity and femininity are well-documented (Bosson et al., 2018). Indeed, males typically report higher masculinity, whereas females report higher femininity (Hsu et al., 2021). This underscores the need to identify which facets of masculinity might facilitate the development of femininity (particularly in males, who often score lower on femininity), and conversely, which facets of femininity might facilitate masculinity (especially in females). Pinpointing these facilitative facets is key to understanding sex-specific pathways to androgyny, which is essential for facilitating a balanced development of both traits within each sex.

Network analysis offers a powerful framework for modeling the complex interconnections among trait facets. In this approach, each facet (e.g., brave and caring) is represented as a “node”, and statistical associations between facets are modeled as “edges” (Mullarkey et al., 2019). A key concept in network analysis is centrality, which quantifies the extent to which a specific facet is connected to all other facets in the network (Liang et al., 2023). Facets with high centrality are considered to exert significant influence within the global network (Borsboom & Cramer, 2013). For example, if “brave” exhibits high centrality, it indicates that it is strongly connected to other facets in the androgynous network. Thus, the centrality metrics are crucial for identifying the most influential facets within the androgynous network.

Another important concept is bridge centrality, which measures how much a node (facet) connects different communities within a network (Liang et al., 2023). Facets with high bridge centrality are considered to act as bridges, facilitating influence between distinct communities within the network (P. J. Jones et al., 2021). For example, if “brave” exhibits high bridge centrality, this indicates that brave has strong connections to facets of femininity (i.e., femininity network) in the androgynous network. Thus, bridge centrality metrics can serve to determine which facets of masculinity function as cross-dimensional facilitators for femininity (especially among males), or which facets of femininity enhance masculinity (especially among females).

Two key metrics for assessing centrality and bridge centrality are node strength and expected influence (EI). Node strength is the sum of the absolute values of a node’s connections (edge weights), ignoring whether the associations are positive or negative (Opsahl et al., 2010). Thus, a facet with high strength has strong overall connections, but its net effect on the network remains ambiguous. In contrast, EI is calculated by summing the signed edge weights (Robinaugh et al., 2016). This means EI accounts for the direction (positive/negative) of each connection, with a higher EI indicating that a facet has a greater net positive impact on the overall network state. In other words, a facet with high EI actively promotes activation and integration within the androgynous network. Bridge strength and bridge EI are analogous calculations, but consider only a facet’s connections to nodes in other predefined communities (e.g., from the masculinity cluster to the femininity cluster) (P. J. Jones et al., 2021). Given our aim to understand facets that promote network activation and integration, this study employs EI and bridge EI as its primary metrics. These measures, by incorporating the direction of associations, provide a more precise gauge of a facet’s net facilitative influence on the global androgynous network.

In summary, this study has three primary aims. First, we will identify the most influential facets within the androgynous network by examining their within-dimensional centrality using EI. Second, we will determine the key cross-dimensional facilitators by analyzing bridge EI to pinpoint which masculinity facets best connect to the femininity network (particularly in males) and which femininity facets best connect to the masculinity network (particularly in females). Finally, we will an exploratorily compare the network structures between males and females to investigate potential sex differences.

## 2. Methods

### 2.1. Participants

Participants were recruited via convenience sampling from multiple classes across several secondary schools in Hefei, China. Eligibility was limited to students enrolled at the selected schools; individuals over 18 were excluded. Although no formal quotas were set, the sample included students of both sexes from various grades. From the initial pool, 112 adolescents were excluded for failing attention-check items (e.g., incorrectly answering “Please select option ‘5’”). The final analytical sample comprised 1270 adolescents (*M*_age_ = 15.41, *SD* = 0.88, age range = 14–17), including 659 males (51.89%, *M*_age_ = 15.44, *SD* = 0.84) and 611 females (*M*_age_ = 15.39, *SD* = 0.92). For more detailed information on participants’ grade levels, parental education level, and perceived family income, see Table S1.

This study was approved by the Ethical Committee for Scientific Research at Hainan University and conducted by the Declaration of Helsinki (2013 Version). Before data collection, class advisors were contacted, and informed consent was obtained from all participants and their legal guardians. The consent form outlined the study’s objectives, confidentiality procedures, and participants’ rights (e.g., the right to withdraw at any time). Participants then received a pencil-and-paper survey packet that included a brief description of the study’s purpose, with emphasis on the exploration of gender norms.

Questionnaires were completed under standardized conditions in a quiet classroom environment, with both researchers and class teachers present. To minimize social desirability and potential influence from authority figures, students returned their completed surveys directly to the researchers. All responses were anonymized during both data collection and processing to ensure participant privacy and confidentiality. As compensation, all participants received an identical pencil.

### 2.2. Measures

#### 2.2.1. Gender-Typed Traits

Masculinity and femininity were assessed by the Chinese Sex-Role Inventory (CSRI) (D.-Z. Liu et al., 2011). It comprises 16 items associated with femininity, 16 items linked to masculinity, and 18 items associated with neutral traits. For this study, which focuses specifically on gender-typed traits, only the 32 masculinity and femininity items were analyzed. Participants rated each facet (e.g., “brave,” “gentle”) on a 7-point scale from 1 (strongly disagree) to 7 (strongly agree), with higher total scores indicating a greater degree of the respective trait. This inventory has demonstrated strong reliability and validity in Chinese samples (W. Liu et al., 2024; Ying et al., 2024). In this study, Cronbach’s alpha was 0.92 for both the masculinity and femininity subscales. The complete list of the 32 items, in both English and Chinese, is provided in Table S2.

#### 2.2.2. Sex

Participants’ sex was determined based on official national identification records, which in the Chinese context correspond to sex assigned at birth (male or female). While international scholarship increasingly emphasizes gender diversity and the inclusion of non-binary identities (Hyde et al., 2019), these concepts are not yet widely reflected in large-scale institutional data collection within Mainland China. The collection of this binary sex information was strictly for the present research. All participants were informed of their right to withdraw from the study at any time, including if they felt discomfort with providing this information.

### 2.3. Statistical Analysis

First, descriptive statistics, correlations, and sex differences for the overall masculinity/femininity scores and the 32 facets were computed using SPSS 27.0. Second, the network analysis was carried out using the R package qgraph (Version 4.4.3) (Epskamp et al., 2012), where nodes represented 32 facets of masculinity and femininity, and edges denoted regularized partial correlations between these facets after controlling for all other facets. Network estimation was based on the graphical Least Absolute Shrinkage and Selection Operator (LASSO) with the Extended Bayesian Information Criterion (EBIC) (Friedman et al., 2010), using a tuning parameter γ = 0.5 to yield a sparse and interpretable structure (Liang et al., 2023). Third, centrality metrics (i.e., EI) were computed using the R package qgraph (Robinaugh et al., 2016). Bridge centrality metrics (i.e., bridge EI) were calculated using the R package networktools (P. J. Jones et al., 2021). Fourth, to assess the accuracy and stability of the estimated network, we employed the R package bootnet (Epskamp et al., 2018). Specifically, nonparametric bootstrapping (1000 iterations) was applied to generate 95% confidence intervals (CIs) for edge weights and to test for significant differences between edges. Additionally, a case-dropping bootstrap procedure was used to evaluate the robustness of centrality estimates, yielding a correlation stability coefficient (CS-coefficient). A CS-coefficient value above 0.25 indicates that centrality indices can be interpreted; values above 0.50 are preferable, and values above 0.70 reflect excellent stability (Epskamp et al., 2018). Finally, to examine sex differences in androgynous network structure, the R package Network Comparison Test (NCT) was employed (van Borkulo et al., 2015). Specifically, the network structure invariance test assesses whether the structure of the two networks differs by comparing the largest edge weights between sexes, applying Bonferroni correction for multiple comparisons to control for Type I error. The global EI invariance test assesses whether the overall net impact, accounting for both positive and negative edge weights, differs between networks. All tests were based on 1000 permutations to ensure robust inference. Network visualization was implemented using the Fruchterman-Reingold algorithm (Fruchterman & Reingold, 1991), which positions highly connected nodes centrally while minimizing node overlap. Positive and negative partial correlations were indicated by green and red edges, respectively.

## 3. Results

### 3.1. Descriptive Statistics, Correlations, and Sex Differences

First, the skewness (−1.11 to 0.35) and kurtosis (−1.22 to 1.48) for the overall masculinity and femininity scores, as well as all individual facets, fell within acceptable ranges.

Second, compared to females, males scored significantly higher than females on overall masculinity (*t* = 9.06, *p* < 0.001, Cohen’s *d* = 0.51) and significantly lower on overall femininity (*t* = −5.47, *p* < 0.001, Cohen’s *d* = −0.31). At the facet level, males scored higher on the majority of masculine facets (13 of 16), while females scored higher on most feminine facets (9 of 16; see Table 1). These results suggest that pathways to androgyny may differ by sex, with males potentially requiring a greater emphasis on developing femininity and females on masculinity.

**Figure 1:**
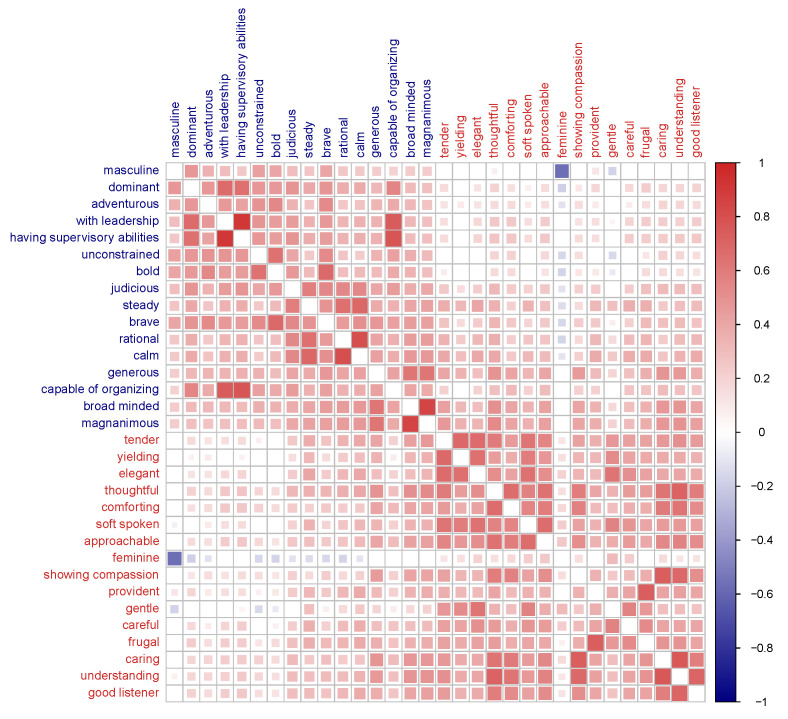
Heatmap of Pearson Correlations for 32 Facets of Masculinity and Femininity. Notes. Correlation strength is indicated by color intensity (blue for negative, red for positive), with significance shown by squares increasing in size from *p* < 0.001 to *p* < 0.05. Variable labels in blue represent 16 facets of masculinity, while those in red represent 16 facets of femininity.

Third, a significant positive correlation was observed between overall masculinity and femininity (*r* = 0.40, *p* < 0.001). As shown in Figure 1, this pattern was reflected at the facet level, with a substantial number of significant positive correlations emerging not only within the same trait category (masculinity–masculinity, femininity–femininity) but also across categories (masculinity–femininity). This pattern of positive intercorrelations provides empirical support for an androgynous structure at the facet level (Figure 1).

### 3.2. Network Structure

**Figure 2:**
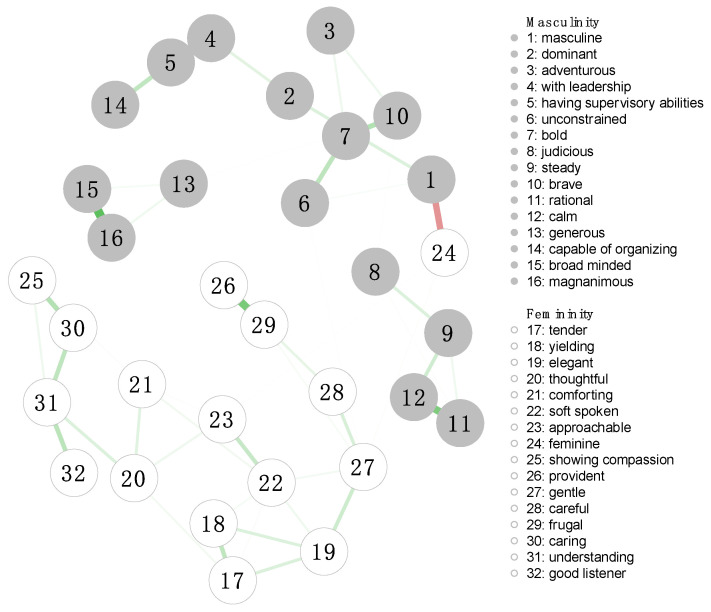
The Androgynous Network Structure in the Full Sample. Notes. The green color of the edges indicates a positive correlation, and red indicates a negative correlation. The thickness of the lines represents the size of their partial correlations. Edges with weights exceeding 0.99 were visually capped, and those with weights below 0.1 were excluded from the graph to ensure a clearer representation of the network structure. The absolute edge weights in this network ranged from 0.10 to 0.68.

**Figure 3:**
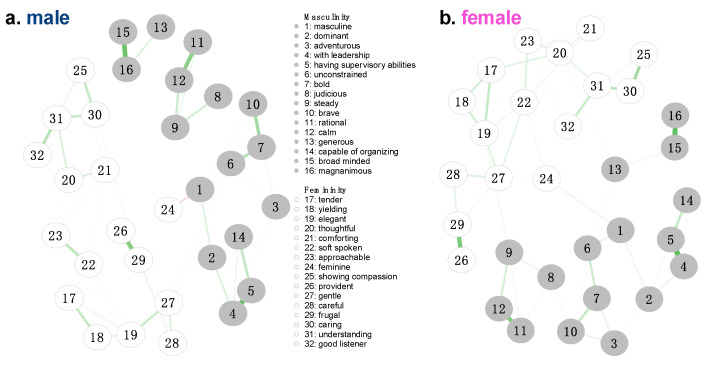
The Androgynous Network Structure in the Male and Female Sample. Notes. The green color of the edges indicates a positive correlation, and red indicates a negative correlation. The thickness of the lines represents the size of their partial correlations. Edges with weights exceeding 0.99 were visually capped, and those with weights below 0.1 were excluded from the graph to ensure a clearer representation of the network structure. The absolute edge weights in this network ranged from 0.10 to 0.64 for males and from 0.10 to 0.67 for females.

The estimated androgynous network, comprising the 32 facets, contained 230 nonzero edges out of a possible 496 (network density = 0.46), indicating a moderately interconnected structure. The average absolute edge weight was 0.22, indicating several strong connections persisted despite the application of regularization (Figure 2). The strongest edge in the network was observed between “leadership” and “supervisory abilities” (*r* = 0.68. All nonzero edges with absolute weights between 0.10 and 0.99 are reported in Table S3. Notably, this strongest edge remained stable across both the male (Figure 3a, Table S4) and female (Figure 3b, Table S5) networks.

### 3.3. EI and Bridge EI Metrics

**Figure 4:**
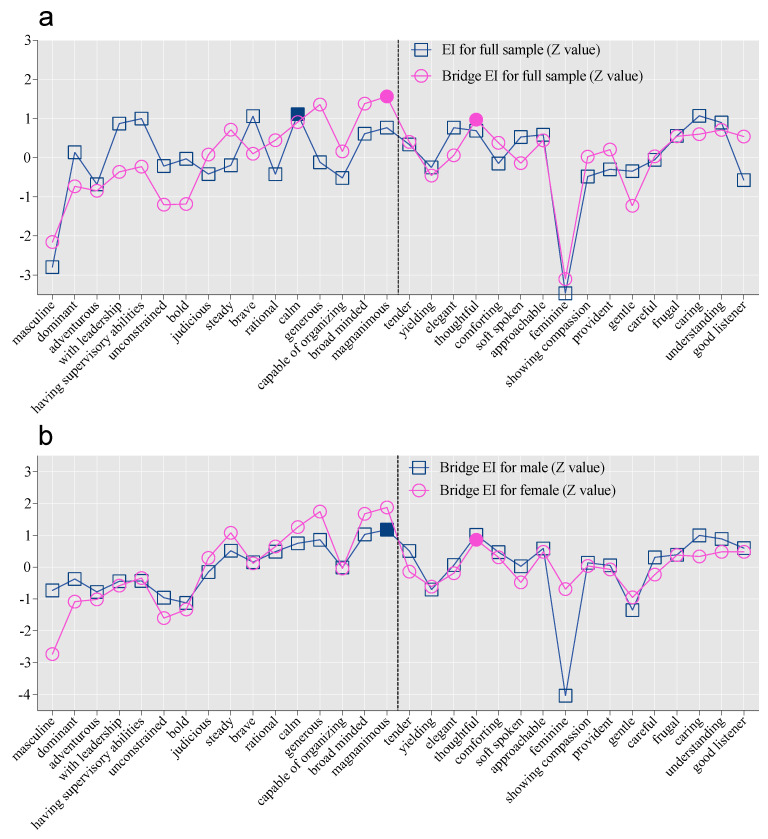
Standardized EI or Bridge EI for each Facet in the Androgynous Network Structure. Notes. Panel a shows EI and bridge EI for the full sample, whereas (**b**) shows bridge EI for males and females only. In (**a**), the blue solid square indicates the facet with the highest EI, and the pink solid square indicates the facets with the highest bridge EI within masculinity (i.e., magnanimous) and femininity (i.e., thoughtful). In (**b**), the blue solid square represents a facet of masculinity (i.e., magnanimous) that exerted the strongest bridging influence on the femininity network in males, and the pink solid circle represents a facet of femininity that exerted the strongest bridging influence on the masculinity network (i.e., thoughtful) in females.

In the full sample, the facet with the highest expected influence (EI) was “calm” (EI = 1.11, Figure 4a). In addition, the facet with the highest bridge EI was “magnanimous” (bridge EI = 1.56), which was the strongest connector to the femininity network. Conversely, the femininity facet “thoughtful” (bridge EI = 0.97) was the strongest connector to the masculinity network.

Analysis of the sex-specific networks revealed distinct pathways. For males, the facet with the highest bridge EI was the masculinity trait “magnanimous” (bridge EI = 1.18, Figure 4b); for females, the facet with the highest bridge EI was also “magnanimous” (bridge EI = 1.88). However, the most influential bridge facet from femininity to masculinity for females was “thoughtful” (bridge EI = 0.86), highlighting its role as a potential facilitator for integrating masculinity in females.

### 3.4. Network Accuracy and Stability

In the full sample, the CS-coefficient for EI was 0.75 (Figure S1). Critically, the CS-coefficient for bridge EI was also 0.75 (Figure S2), confirming the high stability of this key metric. This excellent stability was maintained in the sex-specific networks. The CS-coefficient for bridge EI was 0.75 in both the male (Figure S3) and female (Figure S4) networks.

### 3.5. Network Comparison

The Network Comparison Test (NCT) revealed that the global structure of the androgynous network was invariant between males and females (*M* = 0.16, *p* = 0.488). Furthermore, the edge invariance test found no significant sex differences in specific connections after applying the Holm-Bonferroni correction for multiple comparisons. However, a sex difference emerged in the overall connectivity of the networks (*S* = 0.38, *p* = 0.008), with the female network exhibiting a higher global EI (14.51) than the male network (14.13). This indicates a greater overall level of activation and integration within the female androgynous network.

At the facet level, females showed higher EI than males on the facets “feminine” (EI: *p* = 0.001), “thoughtful” (*p* = 0.022), whereas males showed higher EI than females on “magnanimous” (*p* = 0.043, Table S6). These findings suggest that the relative importance of these facets in promoting androgynous development differs for males and females.

## 4. Discussion

This study used network analysis to examine the facet-level structure of androgyny and to identify sex-specific pathways in androgynous development. For males, the masculinity facet “magnanimous” was the most influential bridge to the femininity network; for females, the femininity facet “thoughtful” was the strongest bridge to the masculinity network. These findings highlight that pathways to androgyny are sex-specific and culturally embedded.

### 4.1. Central Role of “Magnanimous” in Male Androgynous Development

Magnanimous^1^ as an adjective meaning “kind, generous, and forgiving, especially towards an enemy or competitor.” Such characteristics reflect cognitive openness and a capacity for acceptance, suggesting an individual’s capacity to tolerate and reconcile different social expectations. On the one hand, males often exhibit greater cognitive and behavioral rigidity than females, displaying less willingness to adopt new behaviors, particularly those perceived as counter to masculine norms. For example, during the COVID-19 pandemic, males were less likely to adopt preventive measures (Martin & Van Wijk, 2021; S. Jones & Anderson, 2024) and more reluctant to seek healthcare (Wong et al., 2017). The openness inherent in being “magnanimous” could help mitigate the internal conflicts, thereby promoting androgynous development.

On the other hand, feminine males often face lower social acceptance compared to masculine females (Cahill & Adams, 1997; Feinman, 1981; Henry & Steiger, 2022). Girls who display masculine traits are frequently viewed as more competent, whereas boys who exhibit feminine behaviors are often evaluated negatively by their parents (Sandnabba & Ahlberg, 1999) and perceived as having lower social status (Sirin et al., 2004). In this context, the “forgiveness” aspect of magnanimity may provide men with a psychological resource for actively coping with social disapproval.

### 4.2. Central Role of “Thoughtful” in Female Androgynous Development

Thoughtful^2^ as “quiet, because you are thinking/showing that you think about and care for other people/showing signs of careful thought.” Its definition encompasses both introspection and interpersonal consideration. These qualities position “thoughtful” close to masculinity facets such as “calm” and “rational,” while also retaining prototypically feminine attributes such as “careful” and “understanding.” As such, it represents a form of “soft masculinity” that provides females with a natural entry point for integrating agentic traits into their identities.

In the Chinese cultural context, thoughtfulness resonates with Confucian values of benevolence (仁), which emphasize relational harmony. Psychologically, thoughtfulness integrates reflective self-regulation with consideration for others. Developmentally, its centrality suggests “thoughtful” acts as a transitional hub trait, reinforcing culturally valued communal roles while simultaneously facilitating the integration of agentic competencies.

### 4.3. Flexible Gender Development: Cultural Perspectives

These findings can be interpreted within the framework of the gender paradox, where individuals who deviate from prescribed gender roles face psychological and social tension (Berggren & Bergh, 2025; Eagly et al., 2012). Androgynous development, wherein males exhibit femininity and females exhibit masculinity, can create such a conflict (Vescio et al., 2021). However, the identified bridging facets may allow individuals to integrate cross-gender characteristics in a way that minimizes direct violation of societal expectations. Functioning as psychologically proximal facets can facilitate this integration, potentially reducing gender role stress and enabling more flexible self-concepts.

Importantly, these pathways are culturally embedded. For Chinese males, magnanimity provides a socially acceptable way to embrace communal qualities, whereas for females, thoughtfulness facilitates the integration of agentic competencies in line with cultural expectations. This suggests that while gender-typed traits are cultural constructs, they contain inherent, culturally specific opportunities for interconnection. Consequently, pathways to androgyny are likely not universal at the facet levels.

### 4.4. Limitations and Future Research Directions

First, although “magnanimous” and “thoughtful” were central in our Chinese adolescent sample, the key facets facilitating androgyny may differ across cultures. Cross-cultural comparative studies are needed to explore this variability. Second, the current sample primarily included adolescents. However, developmental trajectories of core facets remain unclear. Longitudinal studies across broader age ranges are needed to track how these facets emerge, stabilize, or change. Third, although sex-role inventories like the CSRI provide a structured assessment, they are inherently based on binary gender constructs (Kimmel, 2000). Future studies should aim to incorporate measures that include non-binary identities and reflect greater gender diversity. Fourth, all measures were self-reported, which may introduce bias. Future studies could use more representative samples and include multi-method assessments, such as peer or parent reports.

### 4.5. Practice Implications

The results provide practical insights for psychological and educational interventions. For males, intervention programs designed to enhance psychological flexibility could specifically target the cultivation of magnanimity. For example, using role-playing scenarios that reward acts of kindness and generosity in contexts typically associated with male peer groups (e.g., team sports). This makes the practice of magnanimity socially salient and competence-based, rather than a purely moral injunction. Besides, employ cognitive-behavioral techniques to help boys identify and reframe beliefs that equate kindness with weakness. Exercises could focus on redefining strength to include emotional regulation and prosocial behavior, thereby reducing the cognitive dissonance associated with embracing feminine traits.

For females, empowerment programs could leverage thoughtfulness as a foundation for developing decisiveness and assertiveness. Training that balances communal skills with cognitive control may help females cultivate a more androgynous self-concept. For example, case studies of female leaders could be analyzed to highlight how their thoughtfulness contributed to decisive action, thus bridging the gap between reflection and agency.

## 5. Conclusions

This study demonstrates that androgyny is supported by sex-specific and culturally shaped pathways at the facet level in Chinese adolescents. The masculinity facet “magnanimous” acts as a pivotal facilitator for males, while the femininity facet “thoughtful” serves this function for females. By identifying these bridging traits, the study advances theories of gender development and emphasizes the importance of culturally informed approaches to fostering balanced gender-typed traits. Future research should examine these mechanisms across cultures and extend them to gender-diverse populations.

## Figures and Tables

**Table 1:** Descriptive Statistics (Mean, SD, Skewness, Kurtosis) and Sex Differences in Gender-Typed Trait.

			Males		Females		
Traits and Facets	Skewness	Kurtosis	*M*	*SD*	*M*	*SD*	*p*
Total score of masculinity	−0.20	0.73	5.07	0.97	4.59	0.02	**0.000**
masculine	−0.49	−0.73	5.49	1.33	3.44	1.70	**0.000**
dominant	−0.41	−0.17	4.83	1.45	4.23	1.47	**0.000**
adventurous	−0.28	−0.70	4.75	1.67	4.27	1.66	**0.000**
with leadership	−0.18	−0.42	4.37	1.57	4.24	1.47	0.130
having supervisory abilities	−0.22	−0.42	4.36	1.58	4.29	1.47	0.447
unconstrained	−0.42	−0.38	4.88	1.48	4.51	1.56	**0.000**
bold	−0.30	−0.52	4.81	1.52	4.32	1.52	**0.000**
judicious	−0.72	0.90	5.37	1.22	5.00	1.24	**0.000**
steady	−0.37	−0.17	5.14	1.36	4.55	1.36	**0.000**
brave	−0.43	−0.08	5.19	1.37	4.63	1.37	**0.000**
rational	−0.61	0.07	5.56	1.23	4.92	1.38	**0.000**
calm	−0.58	0.22	5.39	1.25	4.83	1.39	**0.000**
generous	−0.63	0.51	5.38	1.26	5.15	1.27	**0.001**
capable of organizing	−0.35	−0.22	4.62	1.54	4.62	1.38	0.957
broad minded	−0.75	0.65	5.50	1.25	5.20	1.30	**0.000**
magnanimous	−0.75	0.56	5.50	1.27	5.21	1.32	**0.000**
Total score of femininity	−0.38	0.69	4.73	0.98	5.04	1.00	**0.000**
tender	−0.56	−0.11	5.07	1.51	4.87	1.51	**0.020**
yielding	−0.27	−0.61	4.50	1.65	4.36	1.69	0.146
elegant	−0.21	−0.48	4.47	1.59	4.51	1.49	0.630
thoughtful	−0.81	0.77	5.34	1.36	5.48	1.22	0.050
comforting	−0.74	0.03	5.04	1.58	5.31	1.46	**0.002**
soft spoken	−0.29	−0.37	4.54	1.55	4.66	1.52	0.172
approachable	−0.55	0.08	4.82	1.46	5.19	1.38	**0.000**
feminine	0.35	−1.22	1.81	1.32	4.63	1.53	**0.000**
showing compassion	−1.11	1.37	5.47	1.42	5.79	1.18	**0.000**
provident	−0.59	0.14	5.14	1.41	4.96	1.40	**0.026**
gentle	−0.07	−0.69	3.83	1.70	4.32	1.59	**0.000**
careful	−0.21	−0.57	4.31	1.57	4.57	1.57	**0.003**
frugal	−0.47	−0.01	4.90	1.43	4.81	1.42	0.279
caring	−0.92	1.07	5.41	1.31	5.73	1.19	**0.000**
understanding	−1.00	1.48	5.54	1.26	5.70	1.14	**0.024**
good listener	−1.07	1.34	5.56	1.32	5.75	1.21	**0.008**

Notes. Significant *p*-values are in bold.

## Data Availability

The data presented in this study are openly available in the Open Science Framework (OSF) at https://osf.io/udw9y/?view_only=c5ac87c1d3c64d8cb97a011937b62e59 (accessed on 17 August 2025).

## Notes

1 See https://www.oxfordlearnersdictionaries.com/definition/english/magnanimous (accessed on 17 June 2025).

2 See https://www.oxfordlearnersdictionaries.com/definition/english/thoughtful (accessed on 17 June 2025).

## Supplementary Materials

The following supporting information can be downloaded at https://www.mdpi.com/article/10.3390/bs15101375/s1. Figure S1: Stability Analyses of Network Centrality Measures (Strength and Expected Influence) in the Full Sample; Figure S2: Stability Analyses of Network Centrality Measures (Bridge Strength and Bridge Expected Influence) in the Full Sample; Figure S3: Stability Analyses of Network Centrality Measures (Bridge Strength and Bridge Expected Influence) in the Male Sample; Figure S4: Stability Analyses of Network Centrality Measures (Bridge Strength and Bridge Expected Influence) in the Female Sample; Table S1: Descriptive Statistics of Demographic Information for Males and Females; Table S2: Chinese Version and English Version of the Chinese Sex-role Inventory; Table S3: Strengths of the Associations (i.e., Edge Weights) Between Facets Within the Androgynous Network Structure in the Full Sample; Table S4: Strengths of the Associations (i.e., Edge Weights) Between Facets Within the Androgynous Network Structure in the Male Sample; Table S5: Strengths of the Associations (i.e., Edge Weights) Between Facets Within the Androgynous Network Structure in the Female Sample; Table S6: Sex difference in Centrality (Centrality Invariance Test) Within the Masculinity and Femininity Networks.
