# Supplementary material for: Using Network Analysis to Identify Central Facets of Androgynous Development Between Sexes in Chinese Adolescents
Source: Behav Sci (Basel). 2025 Oct 9;15(10):1375. doi: 10.3390/bs15101375 (PMC12562017; doi:10.3390/bs15101375)

Table S1.

Descriptive Statistics of Demographic Information for Males and Females

		Males		Females	
		Frequency	Percentage	Frequency	Percentage
Age	14	109	16.5%	139	22.7%
	15	198	30.0%	143	23.4%
	16	308	46.7%	283	46.3%
	17	44	6.7%	46	7.5%
Father’s education level	1	30	4.6%	24	3.9%
	2	225	34.1%	144	23.6%
	3	174	26.4%	177	29.0%
	4	108	16.4%	102	16.7%
	5	96	14.6%	132	21.6%
	6	26	3.9%	32	5.2%
Mother's education level	1	83	12.6%	47	7.7%
	2	230	34.9%	185	30.3%
	3	174	26.4%	156	25.5%
	4	83	12.6%	112	18.3%
	5	77	11.7%	102	16.7%
	6	12	1.8%	9	1.5%
Perceived family income	1	10	1.5%	8	1.3%
	2	15	2.3%	16	2.6%
	3	38	5.8%	24	3.9%
	4	44	6.7%	44	7.2%
	5	43	6.5%	50	8.2%
	6	52	7.9%	45	7.4%
	7	60	9.1%	61	10.0%
	8	60	9.1%	49	8.0%
	9	50	7.6%	77	12.6%
	10	287	43.6%	245	36.7

*Note***.** Parental education level was coded on a six-point scale: (1) primary school and below, (2) junior high school, (3) senior high school or technical secondary school, (4) junior college, (5) undergraduate degree, and (6) graduate degree. Perceived family income was assessed on a ten-point scale based on monthly household income in Chinese Yuan (CNY): (1) below 2000, (2) 2000–2999, (3) 3000–3999, (4) 4000–4999, (5) 5000–5999, (6) 6000–6999, (7) 7000–7999, (8) 8000–8999, (9) 9000–9999, and (10) above 10000.

Table S2.

Chinese Version and English Version of the Chinese Sex-role Inventory

	Chinese version<br><br>男性气质	English version<br><br>Masculinity	Chinese version<br><br>女性气质	English version<br><br>Femininity
1	乐于冒险的	adventurous	有亲和力的	approachable
2	有领导风范的	with leadership	节俭的	provident
3	大度的	magnanimous	文静的	gentle
4	冷静的	calm	善于倾听的	good listener
5	沉稳的	steady	心细的	careful
6	有判断力的	judicious	能体谅人的	understanding
7	心胸开阔的	broad-minded	语调柔和的	soft-spoken
8	理性的	rational	勤俭的	frugal
9	男子气的	masculine	富有同情心的	showing compassion
10	慷慨的	generous	有爱心的	caring
11	有组织能力的	capable of organizing	乐于安慰人的	comforting
12	胆大的	bold	女子气的	feminine
13	豪放的	unconstrained	文雅的	elegant
14	有领导能力的	having supervisory abilities	温柔的	tender
15	勇敢的	brave	温顺的	yielding
16	有支配力的	dominant	善解人意的	thoughtful

Note. The English version has not been systematically revised to align with the standards for measurement development. This material was originally derived from Ying, J., Shi, J., Liu, S., Endendijk, J. J., & Wu, X. (2024). The Relative Importance of Paternal Versus Maternal Involvement for Chinese Adolescents' Gender-Typed Traits. *Sex Roles*, 90(12), 1866-1878. https://doi.org/10.1007/s11199-024-01531-9

Table S3.

Strengths of the Associations (i.e., Edge Weights) Between Facets Within the Androgynous Network Structure in the Full Sample

Number	Facet 1	Facet 2	Edge weight
1	with leadership	having supervisory abilities	0.68
2	broad minded	magnanimous	0.66
3	provident	frugal	0.57
4	rational	calm	0.55
5	masculine	feminine	-0.48
6	bold	brave	0.37
7	showing compassion	caring	0.37
8	understanding	good listener	0.34
9	unconstrained	bold	0.33
10	caring	understanding	0.33
11	tender	yielding	0.32
12	having supervisory abilities	capable of organizing	0.32
13	soft spoken	approachable	0.30
14	elegant	gentle	0.27
15	steady	calm	0.26
16	gentle	careful	0.24
17	tender	elegant	0.23
18	judicious	steady	0.23
19	yielding	elegant	0.22
20	thoughtful	understanding	0.22
21	masculine	dominant	0.22
22	thoughtful	comforting	0.21
23	dominant	with leadership	0.20
24	careful	frugal	0.19
25	thoughtful	approachable	0.16
26	showing compassion	understanding	0.16
27	adventurous	bold	0.16
28	adventurous	brave	0.16
29	elegant	soft spoken	0.15
30	generous	magnanimous	0.15
31	steady	rational	0.15
32	comforting	soft spoken	0.15
33	tender	thoughtful	0.15
34	soft spoken	gentle	0.14
35	with leadership	capable of organizing	0.14
36	generous	broad minded	0.14
37	yielding	soft spoken	0.14
38	gentle	frugal	0.13
39	masculine	unconstrained	0.12
40	comforting	showing compassion	0.12

41	tender	soft spoken	0.12
42	comforting	approachable	0.11
43	feminine	gentle	0.11
44	judicious	rational	0.11
45	judicious	brave	0.11
46	brave	generous	0.11
47	unconstrained	gentle	-0.11
48	approachable	feminine	0.10
49	adventurous	unconstrained	0.10
50	dominant	adventurous	0.10

Note. The table includes only edge weights with absolute values between 0.10 and 0.99, in line with the visualization thresholds.

Table S4.

Strengths of the Associations (i.e., Edge Weights) Between Facets Within the Androgynous Network Structure in the Male Sample

Number	Facet 1	Facet 2	Edge weight
1	with leadership	having supervisory abilities	0.64
2	broad minded	magnanimous	0.61
3	provident	frugal	0.52
4	rational	calm	0.51
5	bold	brave	0.42
6	unconstrained	bold	0.36
7	understanding	good listener	0.36
8	soft spoken	approachable	0.32
9	having supervisory abilities	capable of organizing	0.32
10	tender	yielding	0.31
11	showing compassion	caring	0.31
12	caring	understanding	0.29
13	elegant	gentle	0.29
14	steady	calm	0.28
15	judicious	steady	0.26
16	thoughtful	comforting	0.23
17	masculine	feminine	-0.22
18	gentle	careful	0.22
19	generous	magnanimous	0.22
20	dominant	with leadership	0.22
21	thoughtful	understanding	0.22
22	masculine	dominant	0.21
23	yielding	elegant	0.20
24	careful	frugal	0.17
25	steady	rational	0.17
26	showing compassion	understanding	0.17
27	with leadership	capable of organizing	0.16
28	tender	elegant	0.16
29	adventurous	brave	0.14
30	comforting	showing compassion	0.14
31	comforting	approachable	0.13
32	frugal	caring	0.13
33	elegant	soft spoken	0.12
34	generous	broad minded	0.12
35	tender	soft spoken	0.12
36	adventurous	with leadership	0.12
37	adventurous	bold	0.12
38	unconstrained	gentle	-0.12
39	comforting	soft spoken	0.12
40	elegant	careful	0.11

41	masculine	brave	0.11
42	adventurous	unconstrained	0.11
43	comforting	understanding	0.11
44	thoughtful	approachable	0.11
45	yielding	soft spoken	0.10
46	magnanimous	thoughtful	0.10
47	tender	approachable	0.10
48	judicious	brave	0.10
49	tender	thoughtful	0.10
50	masculine	unconstrained	0.10

Note. The table includes only edge weights with absolute values between 0.10 and 0.99, in line with the visualization thresholds.

Table S5.

Strengths of the Associations (i.e., Edge Weights) Between Facets Within the Androgynous Network Structure in the Female Sample

Number	Facet 1	Facet 2	Edge weight
1	with leadership	having supervisory abilities	0.67
2	broad minded	magnanimous	0.66
3	provident	frugal	0.56
4	rational	calm	0.55
5	showing compassion	caring	0.41
6	caring	understanding	0.33
7	having supervisory abilities	capable of organizing	0.30
8	tender	elegant	0.30
9	bold	brave	0.30
10	understanding	good listener	0.29
11	unconstrained	bold	0.28
12	tender	yielding	0.26
13	yielding	elegant	0.25
14	steady	calm	0.24
15	gentle	careful	0.23
16	soft spoken	approachable	0.22
17	thoughtful	approachable	0.21
18	elegant	gentle	0.21
19	tender	thoughtful	0.21
20	soft spoken	gentle	0.20
21	careful	frugal	0.20
22	thoughtful	understanding	0.19
23	masculine	unconstrained	0.19
24	adventurous	brave	0.18
25	adventurous	bold	0.18
26	judicious	steady	0.18
27	dominant	with leadership	0.17
28	thoughtful	comforting	0.17
29	masculine	dominant	0.16
30	steady	elegant	0.16
31	elegant	soft spoken	0.16
32	generous	broad minded	0.15
33	masculine	feminine	-0.15
34	comforting	soft spoken	0.15
35	showing compassion	understanding	0.15
36	steady	rational	0.14
37	gentle	frugal	0.14
38	judicious	rational	0.14
39	soft spoken	feminine	0.14
40	with leadership	capable of organizing	0.14

41	yielding	soft spoken	0.13
42	judicious	brave	0.13
43	dominant	having supervisory abilities	0.13
44	feminine	careful	0.12
45	brave	generous	0.12
46	dominant	adventurous	0.11
47	generous	thoughtful	0.11
48	approachable	good listener	0.11
49	yielding	gentle	0.10
50	adventurous	unconstrained	0.10
51	comforting	approachable	0.10
52	comforting	caring	0.10
53	unconstrained	gentle	-0.10

Note. The table includes only edge weights with absolute values between 0.10 and 0.99, in line with the visualization threshold.

Table S6.

Sex difference in Centrality (Centrality Invariance Test) Within the Masculinity and Femininity Networks

Facets	Expected Influence *p*-value
1. feminine	**0.001**
2. thoughtful	**0.022**
3. magnanimous	**0.043**
4. adventurous	0.056
5. brave	0.064
6. frugal	0.075
7. understanding	0.264
8. masculine	0.419
9. unconstrained	0.830
10. steady	0.259
11. having supervisory abilities	0.256
12. bold	0.975
13. yielding	0.148
14. gentle	0.841
15. comforting	0.315
16. dominant	0.070
17. with leadership	0.518
18. judicious	0.721
19. elegant	0.494
20. broad minded	0.052
21. showing compassion	0.866
22. careful	0.504
23. good listener	0.885
24. generous	0.629
25. tender	0.674
26. calm	0.672
27. capable of organizing	0.942
28. rational	0.782
29. caring	0.616
30. soft spoken	0.806
31. provident	0.665
32. approachable	0.995

Note. Significant sex differences are indicated in bold.

Figure S1.

Stability Analyses of Network Centrality Measures (Strength and Expected Influence) in the Full Sample

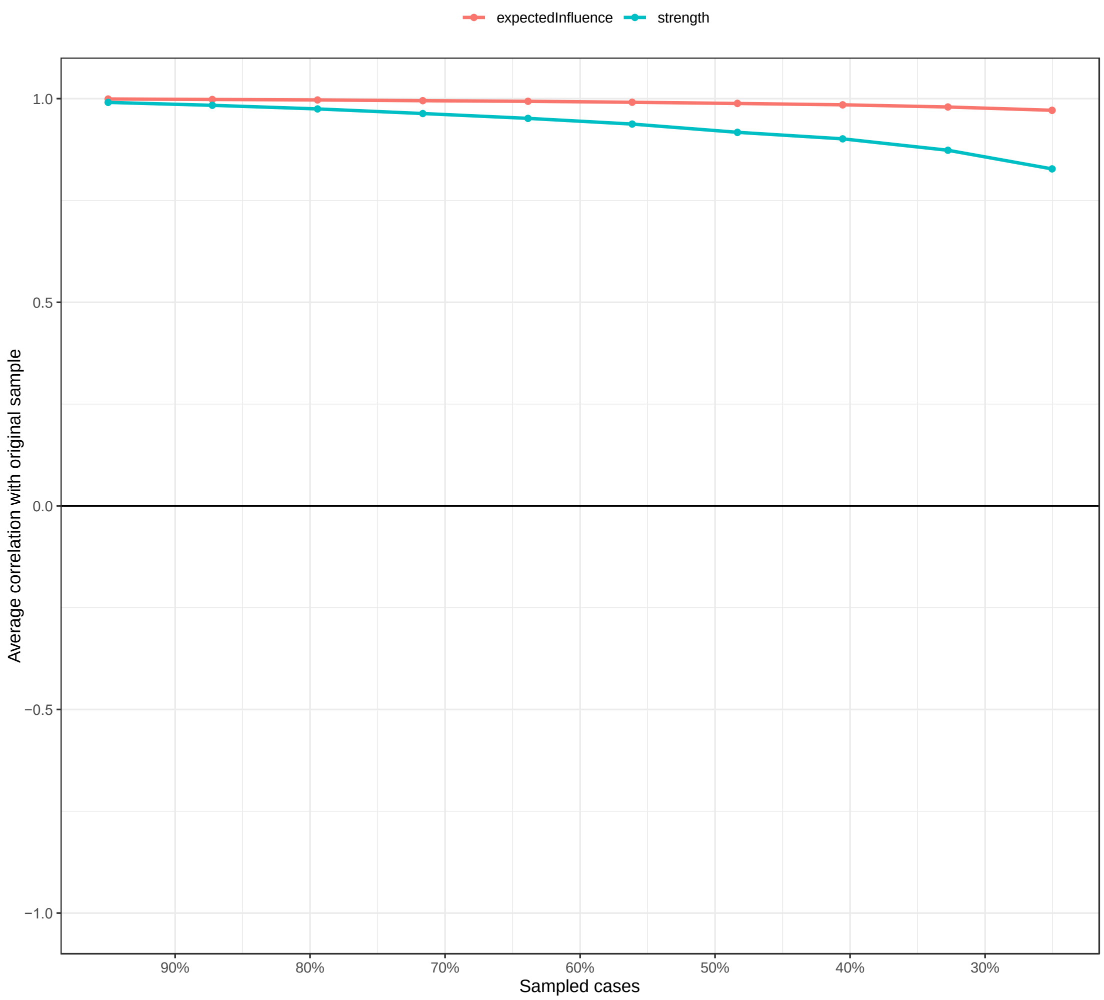

Note. The x-axis represents the percentage of cases from the original sample included at each step. The y-axis represents the average correlations between the original network's centrality indices and those obtained from the networks re-estimated after excluding increasing percentages of cases. Annotations in the following figure are identical.

Figure S2.

Stability Analyses of Network Centrality Measures (Bridge Strength and Bridge Expected Influence) in the Full Sample

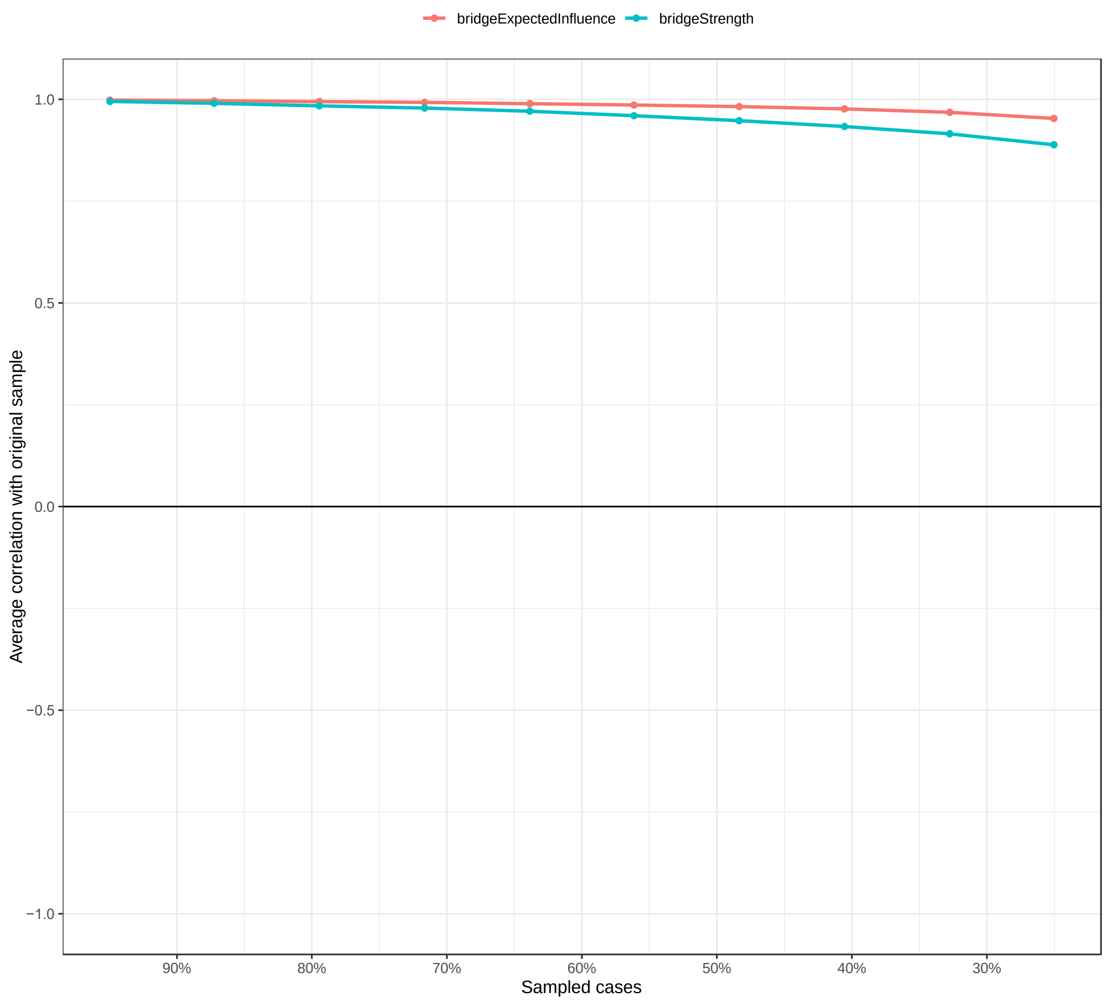

Figure S3.

Stability Analyses of Network Centrality Measures (Bridge Strength and Bridge Expected Influence) in the Male Sample

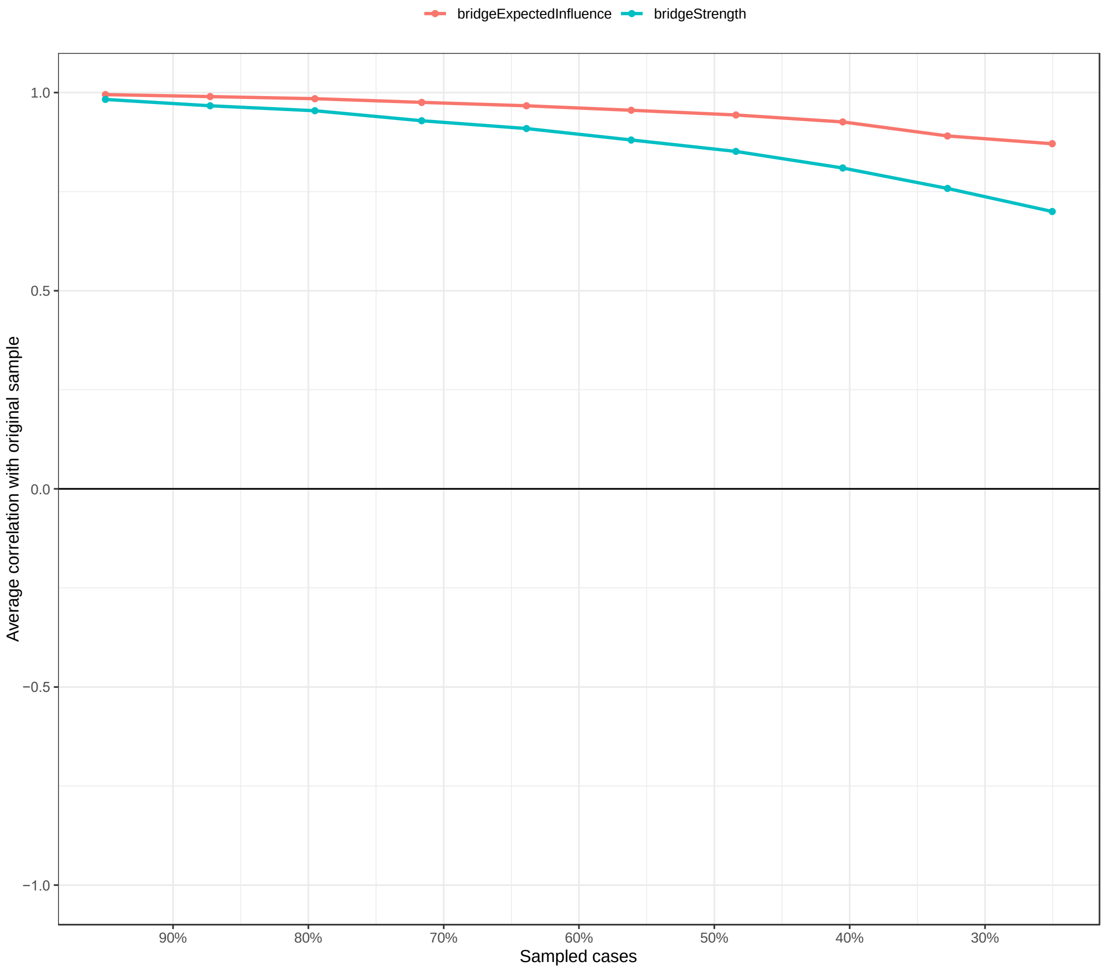

Figure S4.

Stability Analyses of Network Centrality Measures (Bridge Strength and Bridge Expected Influence) in the Female Sample

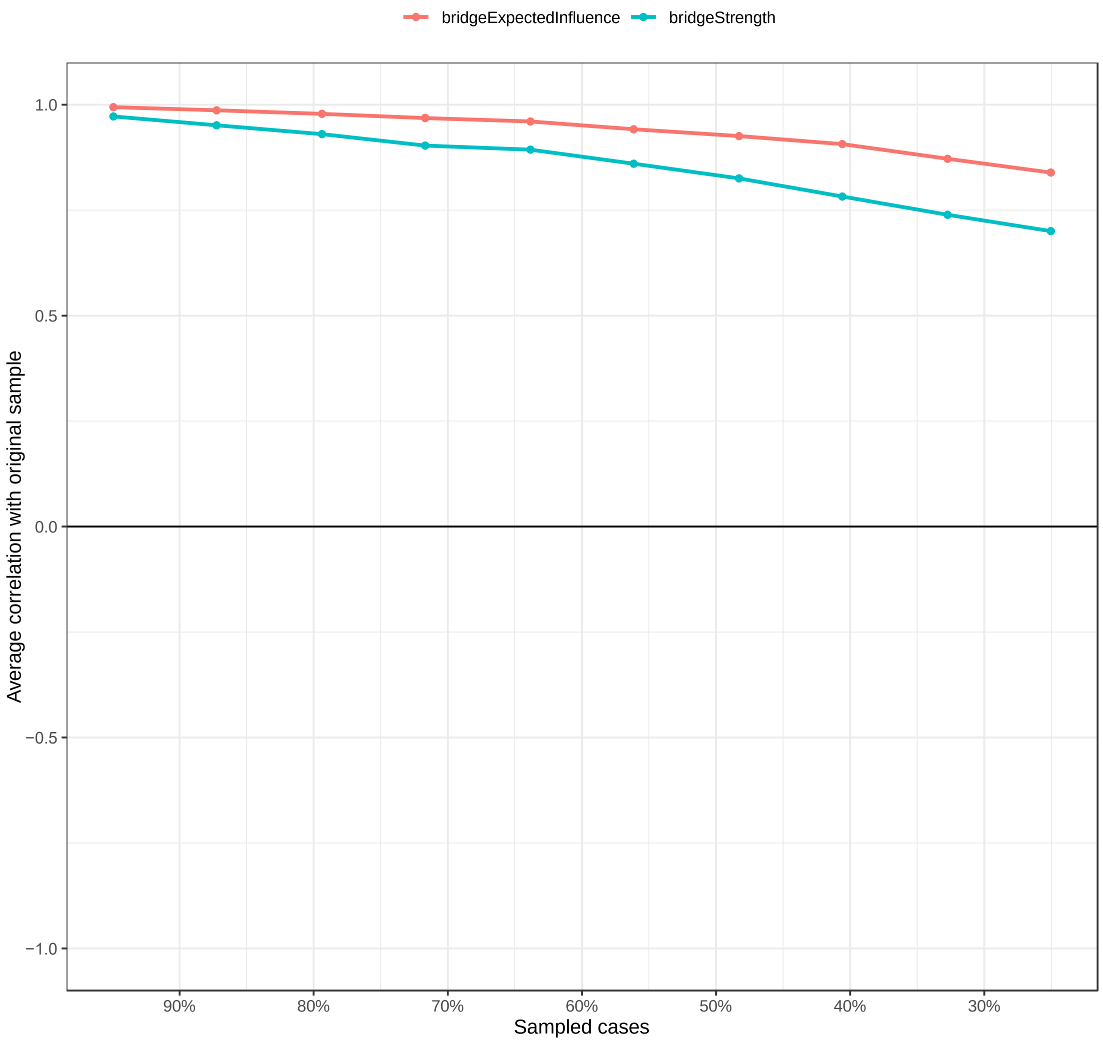

Supplement: Supplementary file 1 [file behavsci-15-01375-s001.zip › behavsci-3855399-supplementary.pdf]
